# Supplementary material for: Computational remodeling of an enzyme conformational landscape for altered substrate selectivity
Source: Nat Commun. 2023 Sep 28;14:6058. doi: 10.1038/s41467-023-41762-0 (PMC10539519; doi:10.1038/s41467-023-41762-0)
Supplement: Supplementary file 2 — Supplementary Information [file 41467_2023_41762_MOESM2_ESM.pdf]

Supplementary Information for

Computational remodeling of an enzyme conformational landscape for altered substrate selectivity

Antony D. St-Jacques,[1,2] Joshua M. Rodriguez,[3] Matthew G. Eason,[1,2] Scott M. Foster,[1,2] Safwat T. Khan,[1,2] Adam M. Damry,[1,2] Natalie K. Goto,[1,2] Michael C. Thompson,[3] Roberto A. Chica[1,2,*]

[1] Department of Chemistry and Biomolecular Sciences, University of Ottawa, Ottawa, Ontario, Canada, K1N 6N5

[2] Center for Catalysis Research and Innovation, University of Ottawa, Ottawa, Ontario, Canada, K1N 6N5

[3] Department of Chemistry and Biochemistry, University of California, Merced, Merced, California 95343, United States

[*] To whom correspondence should be addressed: Roberto A. Chica, Email: rchica@uottawa.ca

This file includes:

Supplementary Tables 1–10

Supplementary Figures 1–16

Supplementary Table 1. Hinge movement analysis for wild–type *E. coli* AAT

DynDom Results	1ARS *vs.* 1ART [a]
**Fixed Domain Residues**	31, 36–324, 339–342, 345–348, 375
**Moving Domain Residues**	13–30, 32–35, 325–338, 343–344, 349–374, 376–394
**Unassigned Residues**	1–12, 395–396
**Angle of rotation (°)**	7.1
**Translation along axis (Å)**	–0.4
**Closure (%)**	100.0
**Bending Residues**	29–32, 35–37, 323–325, 338–349, 374–376

[a] Amino-acid residues are numbered according to the AAT sequence (Uniprot ID: P00509). The 1ARS and 1ART crystal structures of wild-type *E. coli* AAT were used to represent the open and closed states, respectively. For both structures, unit cells of similar dimensions (1ARS: a = 155.42 Å, b = 87.13 Å, c = 79.4 Å; 1ART: a = 157.14 Å, b = 85.51 Å, c = 78.93 Å) corresponding to space group C 2 2 2_1 contain a single protein chain. All analyses performed using DynDom.

Supplementary Table 2. Computed energies of individual mutant sequences from each designed combinatorial library

Closed Library [a]				$Open_{Low}$ Library [a]				$Open_{High}$ Library [a]			
**Mutant** [b]	**E_{closed}** [c] **(kcal mol^{-1})**	**E_{open}** [c] **(kcal mol^{-1})**	**ΔE** [c] **(kcal mol^{-1})**	**Mutant** [b]	**E_{closed}** [c] **(kcal mol^{-1})**	**E_{open}** [c] **(kcal mol^{-1})**	**ΔE** [c] **(kcal mol^{-1})**	**Mutant** [b]	**E_{closed}** [c] **(kcal mol^{-1})**	**E_{open}** [c] **(kcal mol^{-1})**	**ΔE** [c] **(kcal mol^{-1})**
IFIF	–358.9	–272.9	–86.0	IFNH	–387.7	–388.5	0.8	AIFF	–231.5	–359.9	128.4
LFIL	–354.6	–277.2	–77.4	IFCH	–384.1	–385.1	1.1	AIFW	–231.1	–359.4	128.2
LYIL	–350.2	–273.4	–76.8	VFNS	–390.6	–391.8	1.2	CIFM	–239.9	–367.5	127.6
IFIL	–353.3	–280.0	–73.3	VFNA	–386.8	–388.3	1.5	AIFY	–231.2	–358.8	127.6
IFIY	–357.3	–291.6	–65.7	VFNH	–388.6	–391.6	3.0	AIFS	–237.2	–364.4	127.2
VFIY	–357.8	–300.0	–57.8	IFNA	–385.2	–388.6	3.4	AIFM	–235.3	–362.4	127.1
VFIL	–356.6	–298.8	–57.8	IFNS	–388.5	–392.1	3.7	CIFS	–241.7	–368.5	126.9
IYIF	–306.9	–250.2	–56.7	VFCS	–385.6	–389.7	4.2	AIFH	–239.3	–365.5	126.1
VYIL	–350.7	–295.4	–55.3	VFCA	–381.7	–386.3	4.6	SIFF	–232.8	–358.6	125.9
VYIY	–349.5	–294.6	–54.9	VFCH	–384.8	–389.5	4.7	SIFS	–238.1	–363.8	125.7
VYIF	–352.8	–302.4	–50.4	IFCA	–381.3	–387.8	6.5	CIFH	–244.1	–369.4	125.3
IFIT	–361.7	–313.4	–48.3	IFCS	–384.5	–391.2	6.7	SIFY	–232.1	–357.4	125.3
IYIL	–301.5	–253.4	–48.2	MFNA	–378.8	–390.3	11.4	SIFM	–236.8	–362.0	125.2
VFIF	–361.9	–315.7	–46.2	MFNS	–382.8	–394.5	11.7	AIFC	–235.2	–360.1	124.9
VFIT	–366.1	–320.6	–45.5	MFCH	–368.5	–383.9	15.3	AIFA	–237.4	–362.1	124.7
VYIT	–355.8	–315.2	–40.6	MFCS	–379.1	–394.6	15.5	CIFC	–240.0	–364.4	124.3
IYIY	–304.5	–273.2	–31.2	MFCA	–375.0	–390.8	15.7	CIFA	–242.1	–366.3	124.2
LFIF	–321.3	–297.2	–24.2	MFNH	–371.4	–387.8	16.4	CIFW	–238.7	–362.8	124.1
LYIF	–316.6	–295.2	–21.4					SIFH	–240.3	–364.1	123.9
LFIY	–334.7	–320.3	–14.4					SIFC	–236.0	–359.3	123.3
LFIT	–353.9	–341.9	–12.0					SIFA	–238.3	–361.5	123.2
LYIY	–329.8	–318.3	–11.5					CIFF	–236.1	–358.3	122.2
IYIT	–306.4	–294.9	–11.5					SIFW	–236.2	–358.3	122.1
LYIT	–348.9	–339.5	–9.5					CIFY	–236.2	–356.9	120.7

[a] Closed, $Open_{Low}$, and $Open_{High}$ libraries comprise 24, 18, and 24 combinatorial mutant sequences, respectively.
[b] Mutants are named on the basis of the amino-acid identity at designed positions. For example, the VFIY mutant from the Closed library contains Val, Phe, Ile, and Tyr residues at positions 35, 37, 43, and 64, respectively.
[c] Boltzmann-weighted average potential energies (T = 300 K) for the closed (E_{closed}) and open (E_{open}) state ensembles were computed using the Phoenix energy function (Methods). The energy difference (ΔE) corresponds to $E_{closed} – E_{open}$.

Supplementary Table 3. Acceptor substrate concentrations used to measure apparent kinetic parameters reported on Table 1

Enzyme	[α-ketoglutarate] (mM)
**WT**	0.625
**HEX**	0.625
**IYIT**	0.3125
**VFIT**	0.3125
**VFIY**	0.3125
**VYIT**	0.3125
**VYIY**	0.3125
**IFCA**	0.1563
**MFCA**	0.1563
**VFCA**	0.1563
**VFCS**	0.3125
**AIFS**	1.0
**CIFC**	0.625
**CIFS**	1.25
**SIFH**	1.25
**SIFS**	1.25

Supplementary Table 4. Apparent kinetic parameters of *E. coli* AAT and its mutants for transamination of α-ketoglutarate as an acceptor with various amino-acid donors

**Enzyme**	**α-ketoglutarate** [a]			
	K_M (mM)	K_I (mM)	k_{cat} (s^{-1})	k_{cat}/K_M (M^{-1} s^{-1})
**Controls**				
**WT** [b]	0.065 ± 0.005	4.0 ± 0.3	12.2 ± 0.3	190000 ± 20000
**HEX** [b]	0.028 ± 0.003	2.8 ± 0.3	1.83 ± 0.06	65000 ± 7000
**Closed Library**				
**IYIT** [c]	0.26 ± 0.06	1.7 ± 0.4	16 ± 2	60000 ± 20000
**VFIT** [b]	0.050 ± 0.004	2.7 ± 0.2	1.15 ± 0.03	23000 ± 2000
**VFIY** [c]	0.09 ± 0.02	1.6 ± 0.3	20 ± 2	220000 ± 50000
**VYIT** [c]	0.10 ± 0.02	1.3 ± 0.2	43 ± 3	430000 ± 90000
**VYIY** [c]	0.049 ± 0.007	1.6 ± 0.2	26 ± 1	530000 ± 80000
**Open$_{Low}$ Library**				
**IFCA** [c]	0.017 ± 0.001	2.0 ± 0.2	53 ± 1	3100000 ± 200000
**MFCA** [c]	0.0128 ± 0.0008	2.4 ± 0.2	46.3 ± 0.8	3600000 ± 200000
**VFCA** [c]	0.019 ± 0.001	1.8 ± 0.2	56 ± 1	2900000 ± 200000
**VFCS** [b]	0.041 ± 0.006	2.6 ± 0.3	7.9 ± 0.4	190000 ± 30000
**Open$_{High}$ Library**				
**AIFS** [b]	0.17 ± 0.01	4.3 ± 0.3	3.6 ± 0.1	21000 ± 1000
**CIFC** [d]	0.10 ± 0.01	4.8 ± 0.6	2.6 ± 0.1	26000 ± 3000
**CIFS** [d]	0.35 ± 0.04	10 ± 1	0.63 ± 0.03	1800 ± 200
**SIFH** [d]	0.14 ± 0.02	13 ± 3	0.69 ± 0.03	4900 ± 700
**SIFS** [d]	0.14 ± 0.01	14 ± 2	0.67 ± 0.02	4800 ± 500

[a]All experiments were performed in triplicate using a single enzyme batch. Fitting of the kinetic data was done with a rate equation that takes into account substrate inhibition by α-ketoglutarate: $v_0 = (v_{max}[S])/(K_M + [S] + [S]^2/K_I)$. Errors of regression fitting, which represent the absolute measure of the typical distance that each data point falls from the regression line, are provided.
[b] 20 mM of L-aspartate as donor substrate was used to determine apparent kinetic parameters of the α-ketoglutarate acceptor.
[c] 20 mM of L-phenylalanine as donor substrate was used to determine apparent kinetic parameters of the α-ketoglutarate acceptor.
[d] 10 mM of L-aspartate as donor substrate was used to determine apparent kinetic parameters of the α-ketoglutarate acceptor.

Supplementary Table 5. Crystallization conditions

Enzyme [a]	Protein (mg mL^{-1})	$(NH_4)_2SO_4$ (M)
**WT**	5	1.79
**HEX**	10	1.79
**VFIT**	5	1.89
**VFIY**	10	1.79
**VFCS**	15	1.79
**AIFS**	10	1.79

[a] All enzymes were crystallized in a mother liquor containing 100 mM HEPES buffer (pH 7.5), 2% PEG-400, and 20 mM maleate, with varying concentrations of ammonium sulfate as noted. Protein solutions were diluted to the above concentrations with 20 mM potassium phosphate buffer (pH 7.5) containing 2 mM EDTA and 10 µM pyridoxal 5'-phosphate.

Supplementary Table 6. Crystallographic data and refinement statistics for structures at 100 and 278 K

	**WT**	**WT**	**HEX**	**HEX**	**VFIT**	**VFIT**	**VFIY**	**VFIY**	**VFCS**	**VFCS**	**AIFS**	**AIFS**
**Maleate**	(–)	(+)	(–)	(+)	(–)	(+)	(–)	(+)	(–)	(+)	(–)	(+)
**PDB ID**	8E9P	8E9K	8E9J	8E9Q	8E9L	8E9M	8E9N	8E9O	8E9R	8E9S	8E9C	8E9D
**Data collection** [a]												
**Temp. (K)**	278	278	278	278	278	278	278	278	278	278	100	100
**Resolution (Å)**	62.51–2.09	62.16–1.83	124.48–2.09	81.70–1.80	124.38–2.31	124.30–1.76	124.43–1.88	124.31–1.96	124.46–1.90	124.25–2.00	67.54–2.18	61.15–1.37
**Space group**	$P\ 6_3$	$P\ 6_3$	$P\ 6_3$	$P\ 6_3$	$P\ 6_3$	$P\ 6_3$	$P\ 6_3$	$P\ 6_3$	$P\ 6_3$	$P\ 6_3$	$P\ 6_3$	$P\ 6_3$
Cell params.												
**a b c (Å)**	144.33 144.33 81.12	143.55 143.55 81.45	143.74 143.74 81.65	143.39 143.39 81.70	143.62 143.62 81.66	143.53 143.53 81.65	143.67 143.67 81.53	143.52 143.52 81.53	143.63 143.63 81.41	143.47 143.47 81.68	141.63 141.63 80.92	141.22 141.22 81.24
**α β γ (°)**	90 90 120	90 90 120	90 90 120	90 90 120	90 90 120	90 90 120	90 90 120	90 90 120	90 90 120	90 90 120	90 90 120	90 90 120
**Chains per asymm. unit**	2	2	2	2	2	2	2	2	2	2	2	2
**R_{pim}**	0.077 (1.119)	0.073 (0.955)	0.103 (1.096)	0.071 (1.037)	0.182 (1.583)	0.068 (1.076)	0.069 (1.259)	0.092 (0.951)	0.070 (1.017)	0.112 (0.722)	0.059 (0.575)	0.014 (0.399)
**$CC_{1/2}$**	0.995 (0.227)	0.996 (0.348)	0.993 (0.273)	0.994 (0.300)	0.982 (0.295)	0.997 (0.267)	0.997 (0.301)	0.993 (0.309)	0.992 (0.303)	0.993 (0.571)	0.997 (0.470)	1.000 (0.590)
**I/σI**	6.8 (0.4)	6.5 (0.5)	5.9 (0.6)	7.4 (0.8)	3.3 (0.5)	7.4 (0.6)	7.3 (0.5)	6.0 (0.6)	6.1 (0.5)	5.1 (0.7)	8.3 (1.2)	24.9 (1.7)
**Complete. (%)**	100.0 (100.0)	100.0 (100.0)	99.8 (99.7)	99.8 (99.3)	100.0 (100.0)	100.0 (100.0)	98.7 (97.6)	100.0 (99.4)	99.8 (98.7)	100.0 (99.1)	100.0 (100.0)	97.7 (92.2)
**Multiplicity**	10.5 (9.3)	10.5 (10.7)	10.2 (10.1)	5.0 (4.8)	10.3 (10.3)	10.0 (9.0)	10.3 (10.5)	10.1 (10.2)	81.5 (82.7)	40.6 (40.5)	40.6 (38.6)	19.8 (17.3)
**Wilson B-factor ($Å^2$)**	31.230	23.930	28.090	23.300	27.750	21.810	26.890	23.820	23.940	21.990	35.942	16.485
**# unique reflections**	57093 (2848)	84225 (4169)	56805 (2782)	88385 (4395)	42181 (2069)	94818 (4736)	76900 (3787)	68674 (3426)	75209 (3696)	64724 (3199)	48326 (2387)	188377 (8826)
**Refinement**												
**R work/free**	0.1743/ 0.2055	0.1528/ 0.1832	0.1675/ 0.1975	0.1435/ 0.1721	0.1829/ 0.2218	0.1477/ 0.1753	0.1505/ 0.1783	0.1481/ 0.1886	0.1528/ 0.1910	0.1479/ 0.1850	0.1891/ 0.2236	0.1487/ 0.1604
No. atoms												
**Protein**	6359	6500	6151	6657	6121	6382	6412	6250	6229	6175	6002	6522
**Ligand**	41	46	42	46	42	46	42	46	41	46	40	46
**Water**	184	389	244	437	157	354	330	377	376	343	172	744
Averaged B-factors ($Å^2$)												
**Protein**	47.22	36.58	40.90	34.25	44.08	33.71	39.07	36.83	38.93	34.33	48.41	22.73
**Ligand**	38.82	26.19	47.31	24.68	43.05	22.71	35.15	27.55	33.19	21.09	47.62	18.10
**Water**	44.24	43.34	41.00	44.32	38.45	42.26	45.19	43.19	46.35	38.95	42.04	32.01
RMSD												
**bond lengths (Å)**	0.002	0.005	0.002	0.006	0.003	0.005	0.007	0.009	0.005	0.005	0.002	0.012
**bond angles (°)**	0.486	0.812	0.495	0.898	0.500	0.781	0.839	0.899	0.793	0.839	0.535	1.224
Molprobity statistics												
**Ramachand. outliers (%)**	0.00	0.00	0.00	0.00	0.00	0.00	0.00	0.00	0.00	0.00	0.00	0.00
**Ramachand. allowed (%)**	3.27	2.39	2.77	2.64	3.03	1.90	2.39	2.14	2.27	2.02	3.38	2.90
**Ramachand. favored (%)**	96.73	97.61	97.23	97.36	96.97	98.10	97.61	97.86	97.73	97.98	96.62	97.10
**Rotamer outliers (%)**	0.61	0.74	1.11	1.72	0.64	1.36	1.36	1.25	1.25	0.94	0.33	0.15
**MolProbity clashscore**	0.64	1.56	0.74	1.43	1.24	1.03	1.81	1.46	2.03	1.23	1.60	3.16

[a] Highest resolution shell is shown in parentheses.

Supplementary Table 7. Hinge movement analysis for AAT variants crystallized here

DynDom Results	WT (8E9P *vs.* 8E9K) [a]	VFCS (8E9R *vs.* 8E9S) [a]	AIFS (8E9C *vs.* 8E9D) [a]
**Fixed Domain Residues**	36–320, 340–342, 345–348	36–318, 340–348	30–318, 339–348
**Moving Domain Residues**	21–32, 321–339, 343–344, 349–394	25–35, 319–339, 349–394	29, 319–338, 349–394
**Unassigned Residues**	1–20, 33–35, 395–396	1–24, 395–396	1–28, 395–396
**Angle of rotation (°)**	4.6	2.6	5.9
**Translation along axis (Å)**	–0.3	–0.2	–0.3
**Closure (%)**	92.5	96.0	92.8
**Bending Residues** [b]	30–38, 320–321, 338–349	35–36, 318–319, 339–341, 343–352	29–46, 318–319, 338–341, 348–352

[a] For WT, VFCS, and AIFS, domain movement between the open and closed states was observed for chain A. Hinge movement was not detected for HEX, VFIT, or VFIY. All analyses performed using DynDom.

Supplementary Table 8. Conformational equilibrium constants of AAT variants at various temperatures

	K_{eq} [a]						
**Enzyme**	**278 K**	**283 K**	**288 K**	**283 K**	**298 K**	**303 K**	**308 K**
**HEX**	24.093	9.047	4.301	3.139	2.190	1.730	1.482
**VFIT**	3.002	1.380	0.790	0.494	0.368	0.279	0.231
**VFIY**	2.058	0.810	0.425	0.219	0.124	0.066	0.046
**AIFS**	0.157	0.521	0.795	1.570	2.308	2.715	12.735

[a] Equilibrium constants were calculated from the ratio of peaks observed by ^{19}F NMR at various temperatures (Supplementary Figure 13). For HEX, VFIT, and VFIY, equilibrium constants are reported for the closed/open conformational transition (K_{eq} = closed/open). For AIFS, equilibrium constants are reported for conformational states corresponding to alternate open conformations.

Supplementary Table 9. Crystallographic data and refinement statistics for structures at 303 K

	**WT**	**HEX**	**VFIT**
**Maleate**	(–)	(–)	(–)
**PDB ID**	8E9T	8E9U	8E9V
**Data collection** [a]			
**Temp. (K)**	303	303	303
**Resolution (Å)**	124.73–2.13	124.43–1.94	124.71–2.01
**Space group**	P 6_3	P 6_3	P 6_3
Cell params.			
**a b c (Å)**	144.03 144.03 81.26	143.66 143.66 81.54	144.01 144.01 81.58
**α β γ (°)**	90 90 120	90 90 120	90 90 120
**Chains per asymm. unit**	2	2	2
**R_{pim}**	0.091 (0.993)	0.093 (1.860)	0.083 (0.823)
**$CC_{1/2}$**	0.993 (0.263)	0.997 (0.334)	0.993 (0.357)
**I/σI**	6.0 (0.5)	7.0 (0.6)	5.5 (0.5)
**Complete. (%)**	100.0 (100.0)	99.3 (98.7)	100.0 (100.0)
**Multiplicity**	5.1 (4.8)	10.2 (10.4)	5.0 (4.9)
**Wilson B-factor (Å^2)**	30.940	29.110	27.850
**# unique reflections**	53790 (2679)	70417 (3433)	64155 (3220)
**Refinement**			
**R work/free**	0.1715/ 0.1997	0.1473/ 0.1776	0.1684/ 0.1961
No. atoms			
**Protein**	6186	6275	6197
**Ligand**	41	42	42
**Water**	175	265	223
Averaged B-factors (Å^2)			
**Protein**	47.39	43.29	41.92
**Ligand**	39.50	43.88	35.10
**Water**	45.51	47.10	44.01
RMSD			
**bond lengths (Å)**	0.002	0.007	0.002
**bond angles (°)**	0.504	1.013	0.517
Molprobity statistics			
**Ramachand. outliers (%)**	0.00	0.00	0.00
**Ramachand. allowed (%)**	2.28	2.40	2.41
**Ramachan. favored (%)**	97.72	97.60	97.59
**Rotamer outliers (%)**	1.26	1.70	0.90
**MolProbity clashscore**	1.15	1.85	1.57

[a] Highest resolution shell is shown in parentheses.

Supplementary Table 10. Amino-acid sequences of AAT variants

Enzyme	# mutations from WT	# mutations from HEX	Sequence[a]
**WT**	–	6	MAHHHHHHVGTFENITAAPADPILGLADLFRADERPGKINLGIGVYKDETGKTPVLTSVK<br>KAEQYLLENETTKNYLGIDGIPEFGRCTQELLFGKGSALINDKRARTAQTPGGTGALRVA<br>ADFLAKNTSVKRVWVSNPSWPNHKSVFNSAGLEVREYAYYDAENHTLDFDALINSLNEAQ<br>AGDVVLFHGCCHNPTGIDPTLEQWQTLAQLSVEKGWLPLFDFAYQGFARGLEEDAEGLRA<br>FAAMHKELIVASSYSKNFGLYNERVGACTLVAADSETVDRAFSQMKAAIRANYSNPPAHG<br>ASVVATILSNDALRAIWEQELTDMRQRIQRMRQLFVNTLQEKGANRDFSFIIKQNGMFSF<br>SGLTKEQVLRLREEFGVYAVASGRVNVAGMTPDNMAPLCEAIVAVL
**HEX**	6	–	MAHHHHHHVGTFENITAAPADPILGLADLFRADERPGKINLGIG**L**Y**Y**DETGK**I**PVLTSVK<br>KAEQYLLENETTK**L**YLGIDGIPEFGRCTQELLFGKGSALINDKRARTAQTPGG**S**GALRVA<br>ADFLAKNTSVKRVWVSNPSWPNHKSVFNSAGLEVREYAYYDAENHTLDFDALINSLNEAQ<br>AGDVVLFHGCCHNPTGIDPTLEQWQTLAQLSVEKGWLPLFDFAYQGFARGLEEDAEGLRA<br>FAAMHKELIVASSYSKNFGLYNERVGACTLVAADSETVDRAFSQMKAAIRANYS**S**PPAHG<br>ASVVATILSNDALRAIWEQELTDMRQRIQRMRQLFVNTLQEKGANRDFSFIIKQNGMFSF<br>SGLTKEQVLRLREEFGVYAVASGRVNVAGMTPDNMAPLCEAIVAVL
**VFIT**	3	5	MAHHHHHHVGTFENITAAPADPILGLADLFRADERPGKINLGIGVY**F**DETGK**I**PVLTSVK<br>KAEQYLLENETTK**T**YLGIDGIPEFGRCTQELLFGKGSALINDKRARTAQTPGGTGALRVA<br>ADFLAKNTSVKRVWVSNPSWPNHKSVFNSAGLEVREYAYYDAENHTLDFDALINSLNEAQ<br>AGDVVLFHGCCHNPTGIDPTLEQWQTLAQLSVEKGWLPLFDFAYQGFARGLEEDAEGLRA<br>FAAMHKELIVASSYSKNFGLYNERVGACTLVAADSETVDRAFSQMKAAIRANYSNPPAHG<br>ASVVATILSNDALRAIWEQELTDMRQRIQRMRQLFVNTLQEKGANRDFSFIIKQNGMFSF<br>SGLTKEQVLRLREEFGVYAVASGRVNVAGMTPDNMAPLCEAIVAVL
**VFIY**	3	5	MAHHHHHHVGTFENITAAPADPILGLADLFRADERPGKINLGIGVY**F**DETGK**I**PVLTSVK<br>KAEQYLLENETTK**Y**YLGIDGIPEFGRCTQELLFGKGSALINDKRARTAQTPGGTGALRVA<br>ADFLAKNTSVKRVWVSNPSWPNHKSVFNSAGLEVREYAYYDAENHTLDFDALINSLNEAQ<br>AGDVVLFHGCCHNPTGIDPTLEQWQTLAQLSVEKGWLPLFDFAYQGFARGLEEDAEGLRA<br>FAAMHKELIVASSYSKNFGLYNERVGACTLVAADSETVDRAFSQMKAAIRANYSNPPAHG<br>ASVVATILSNDALRAIWEQELTDMRQRIQRMRQLFVNTLQEKGANRDFSFIIKQNGMFSF<br>SGLTKEQVLRLREEFGVYAVASGRVNVAGMTPDNMAPLCEAIVAVL
**VFCS**	3	6	MAHHHHHHVGTFENITAAPADPILGLADLFRADERPGKINLGIGVY**F**DETGK**C**PVLTSVK<br>KAEQYLLENETTK**S**YLGIDGIPEFGRCTQELLFGKGSALINDKRARTAQTPGGTGALRVA<br>ADFLAKNTSVKRVWVSNPSWPNHKSVFNSAGLEVREYAYYDAENHTLDFDALINSLNEAQ<br>AGDVVLFHGCCHNPTGIDPTLEQWQTLAQLSVEKGWLPLFDFAYQGFARGLEEDAEGLRA<br>FAAMHKELIVASSYSKNFGLYNERVGACTLVAADSETVDRAFSQMKAAIRANYSNPPAHG<br>ASVVATILSNDALRAIWEQELTDMRQRIQRMRQLFVNTLQEKGANRDFSFIIKQNGMFSF<br>SGLTKEQVLRLREEFGVYAVASGRVNVAGMTPDNMAPLCEAIVAVL
**AIFS**	4	6	MAHHHHHHVGTFENITAAPADPILGLADLFRADERPGKINLGIG**A**Y**I**DETGK**F**PVLTSVK<br>KAEQYLLENETTK**S**YLGIDGIPEFGRCTQELLFGKGSALINDKRARTAQTPGGTGALRVA<br>ADFLAKNTSVKRVWVSNPSWPNHKSVFNSAGLEVREYAYYDAENHTLDFDALINSLNEAQ<br>AGDVVLFHGCCHNPTGIDPTLEQWQTLAQLSVEKGWLPLFDFAYQGFARGLEEDAEGLRA<br>FAAMHKELIVASSYSKNFGLYNERVGACTLVAADSETVDRAFSQMKAAIRANYSNPPAHG<br>ASVVATILSNDALRAIWEQELTDMRQRIQRMRQLFVNTLQEKGANRDFSFIIKQNGMFSF<br>SGLTKEQVLRLREEFGVYAVASGRVNVAGMTPDNMAPLCEAIVAVL

[a] Sequence for WT was obtained from Uniprot (P00509). Mutations from wild-type (WT) AAT are highlighted in bold and underlined. All sequences contain a His-tag at the N-terminus.

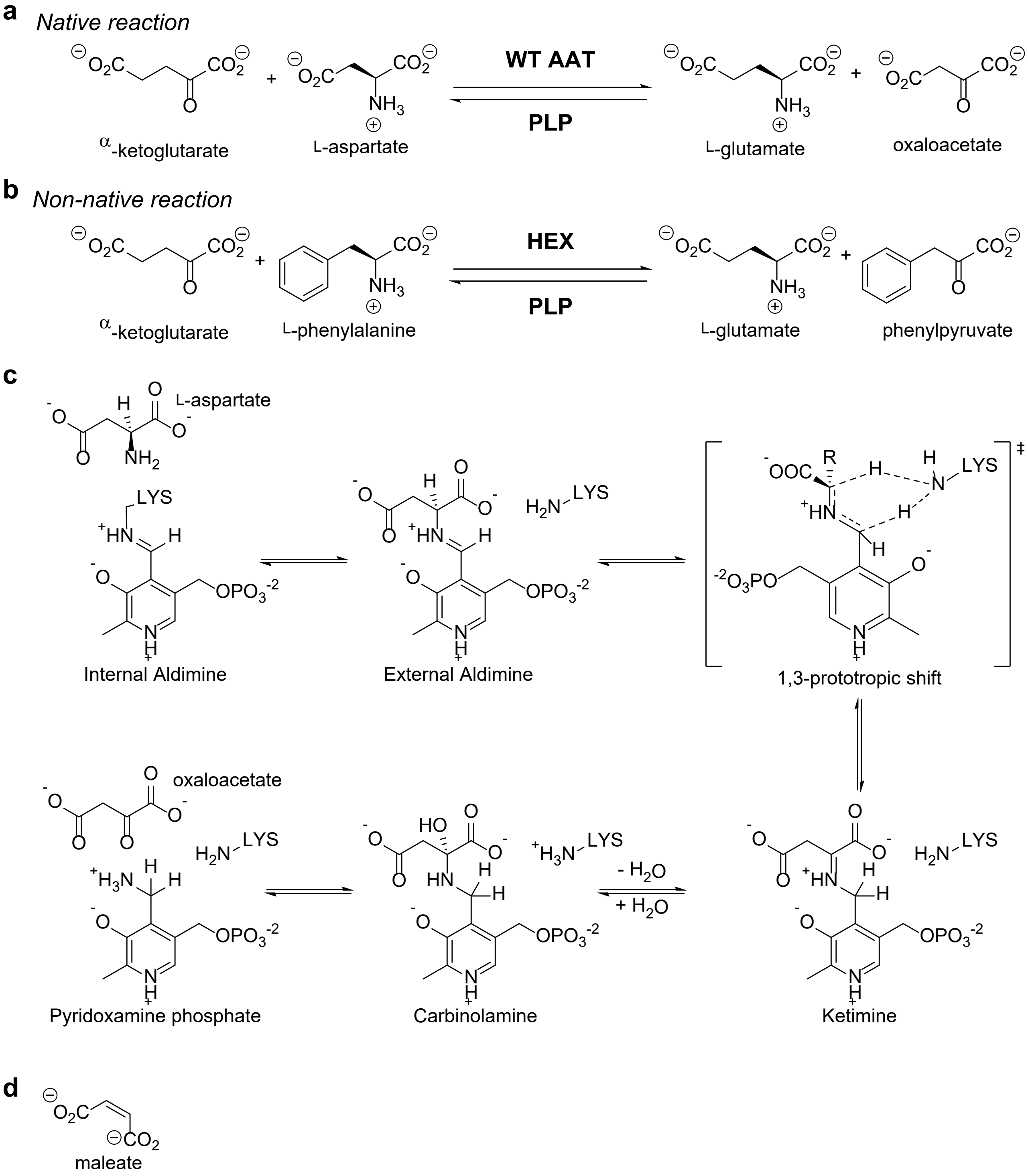

Supplementary Figure 1. Transamination reaction catalyzed by *E. coli* aspartate aminotransferase (AAT). (a) Wild-type (WT) AAT natively catalyzes the reversible transamination of dicarboxylate substrates using the pyridoxal 5'-phosphate (PLP) cofactor. (b) The AAT hexamutant (HEX) can also efficiently catalyze transamination of the non-native substrate L-phenylalanine. (c) Mechanism of the L-aspartate half-reaction of AAT. (d) AAT is inhibited by L-aspartate analogue maleate.

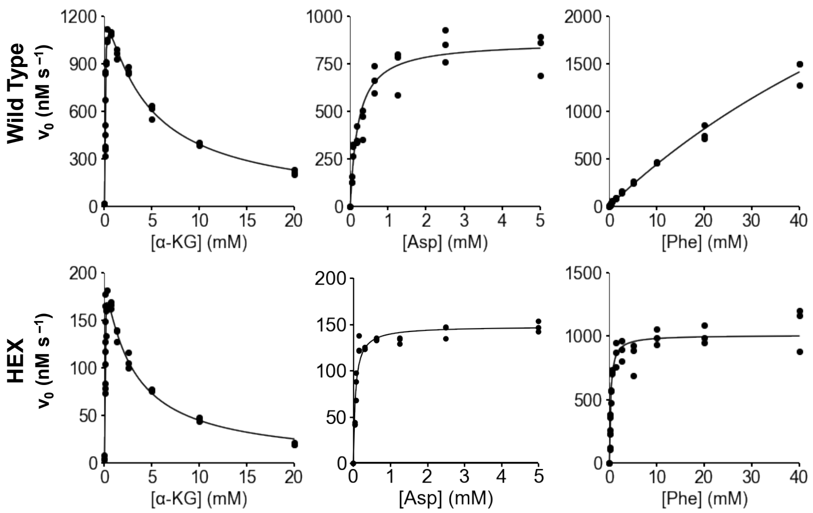

Supplementary Figure 2. Steady-state kinetics of wild-type AAT and HEX. Michaelis-Menten plots of initial rates (normalized to enzyme quantity) as a function of substrate concentrations are shown. α-KG, Asp, and Phe indicate α-ketoglutarate, L-aspartate, and L-phenylalanine, respectively. All experiments were performed in triplicate. For α-KG, fitting of the kinetic data was done with a rate equation that takes into account substrate inhibition (Methods).

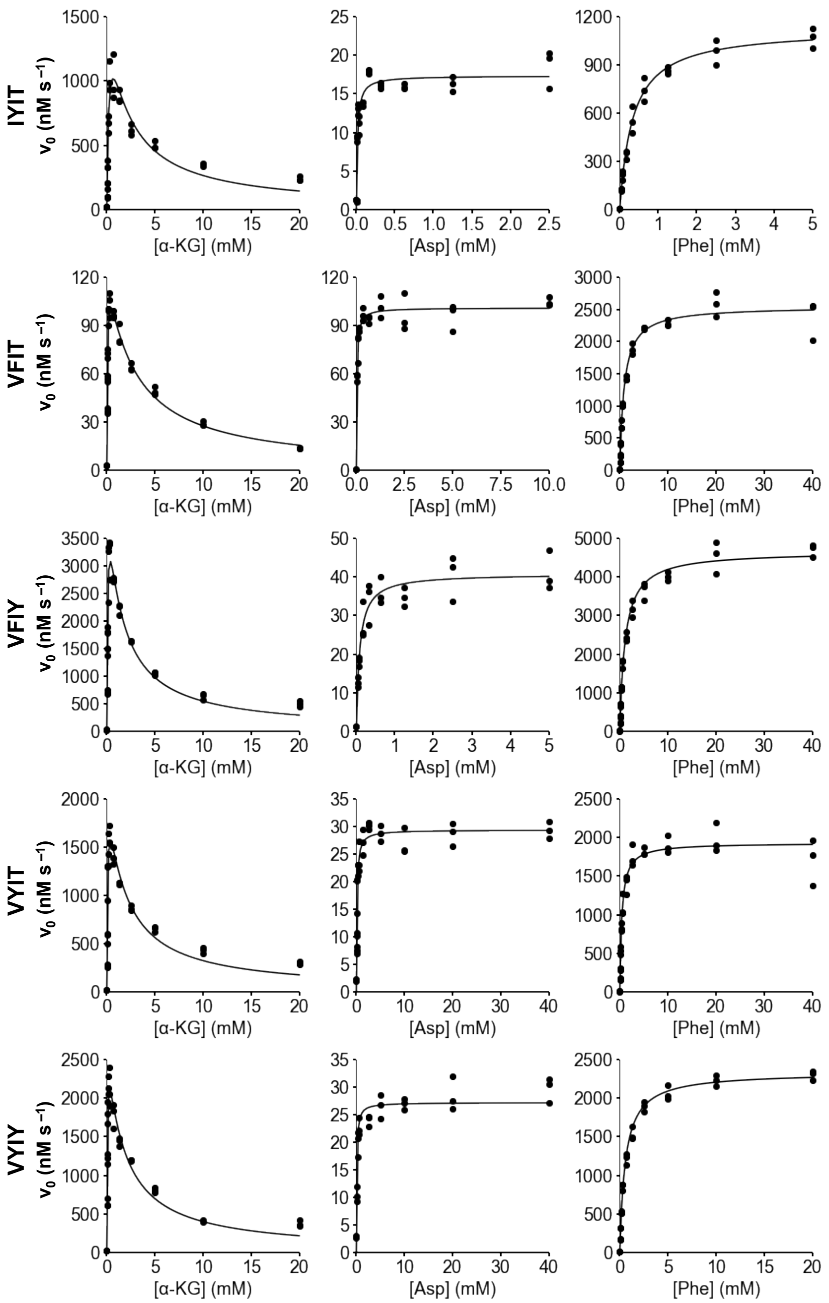

Supplementary Figure 3. Steady-state kinetics of Closed library mutants. Michaelis-Menten plots of initial rates (normalized to enzyme quantity) as a function of substrate concentrations are shown. α-KG, Asp, and Phe indicate α-ketoglutarate, L-aspartate, and L-phenylalanine, respectively. All experiments were performed in triplicate. For α-KG, fitting of the kinetic data was done with a rate equation that takes into account substrate inhibition (Methods).

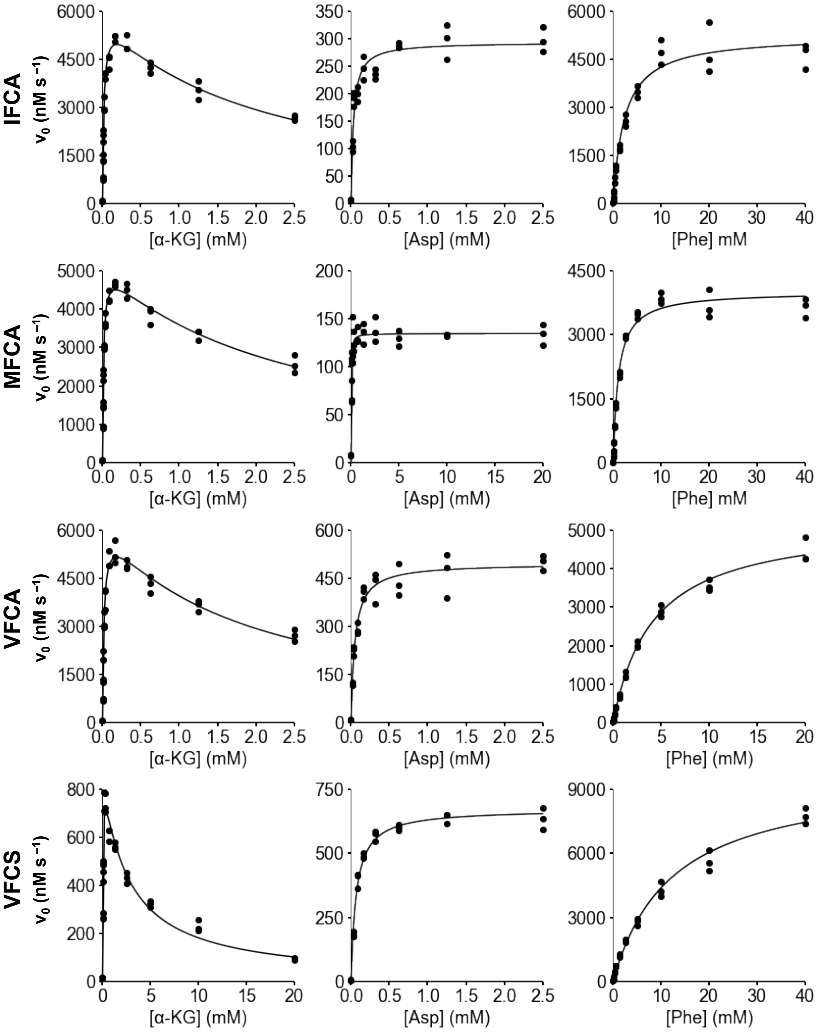

Supplementary Figure 4. Steady-state kinetics of Open$_{\text{Low}}$ library mutants. Michaelis-Menten plots of initial rates (normalized to enzyme quantity) as a function of substrate concentrations are shown. α-KG, Asp, and Phe indicate α-ketoglutarate, L-aspartate, and L-phenylalanine, respectively. All experiments were performed in triplicate. For α-KG, fitting of the kinetic data was done with a rate equation that takes into account substrate inhibition (Methods).

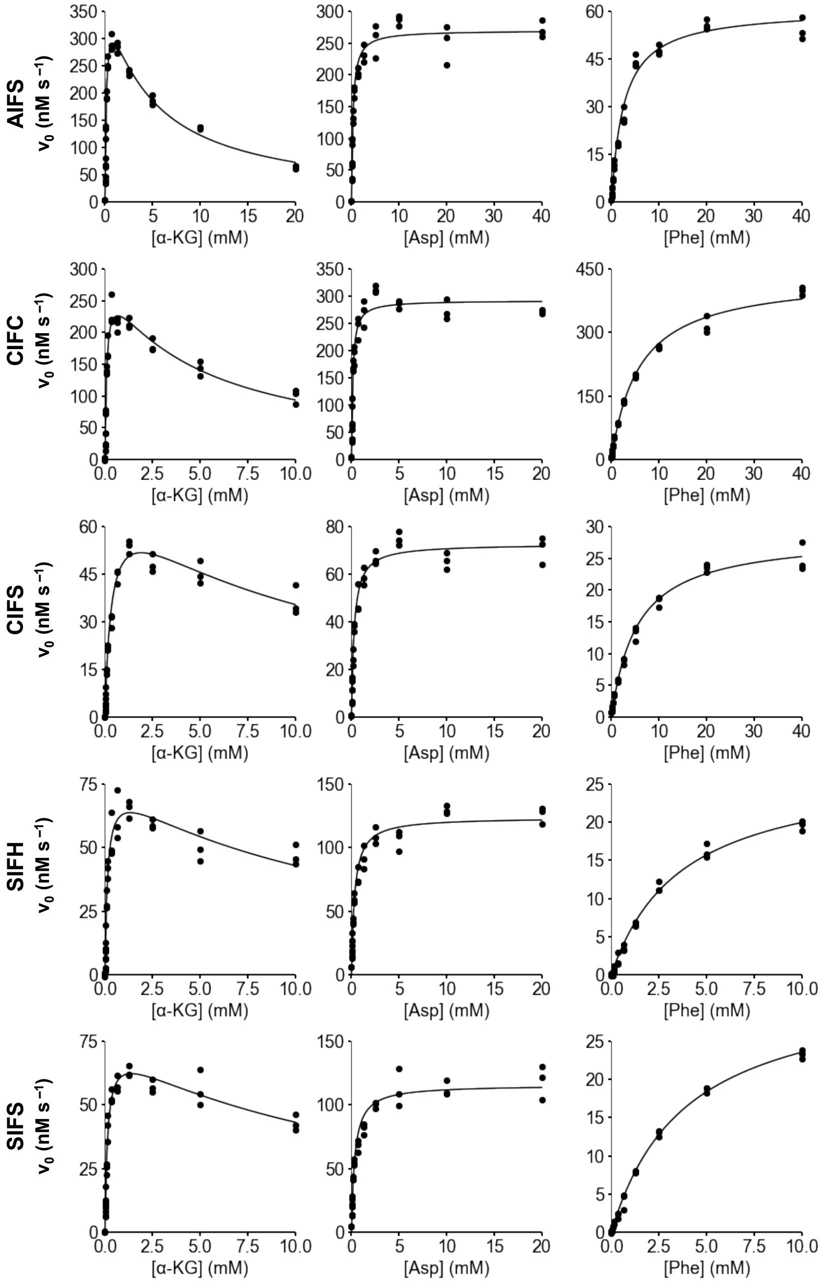

Supplementary Figure 5. Steady-state kinetics of Open$_{High}$ library mutants. Michaelis-Menten plots of initial rates (normalized to enzyme quantity) as a function of substrate concentrations are shown. α-KG, Asp, and Phe indicate α-ketoglutarate, L-aspartate, and L-phenylalanine, respectively. All experiments were performed in triplicate. For α-KG, fitting of the kinetic data was done with a rate equation that takes into account substrate inhibition (Methods).

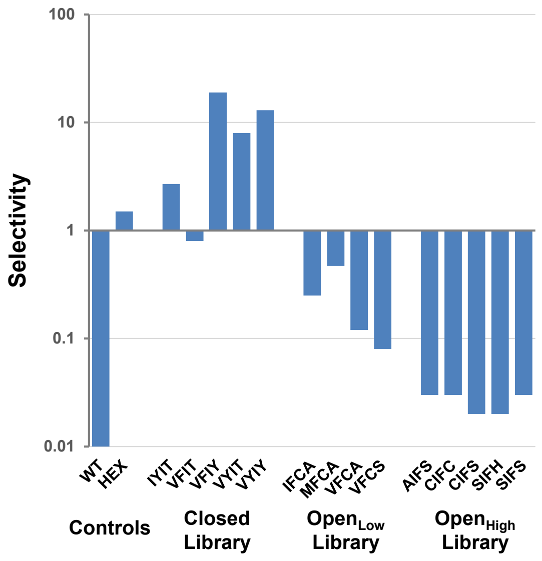

Supplementary Figure 6. Substrate selectivity of AAT variants. Selectivity is defined as (k_{cat}/K_M L-phenylalanine) / (k_{cat}/K_M L-aspartate). Although Closed Library mutant VFIT has a selectivity value of 0.8, it is approximately 60-fold more active with the non-native substrate than the wild type (WT).

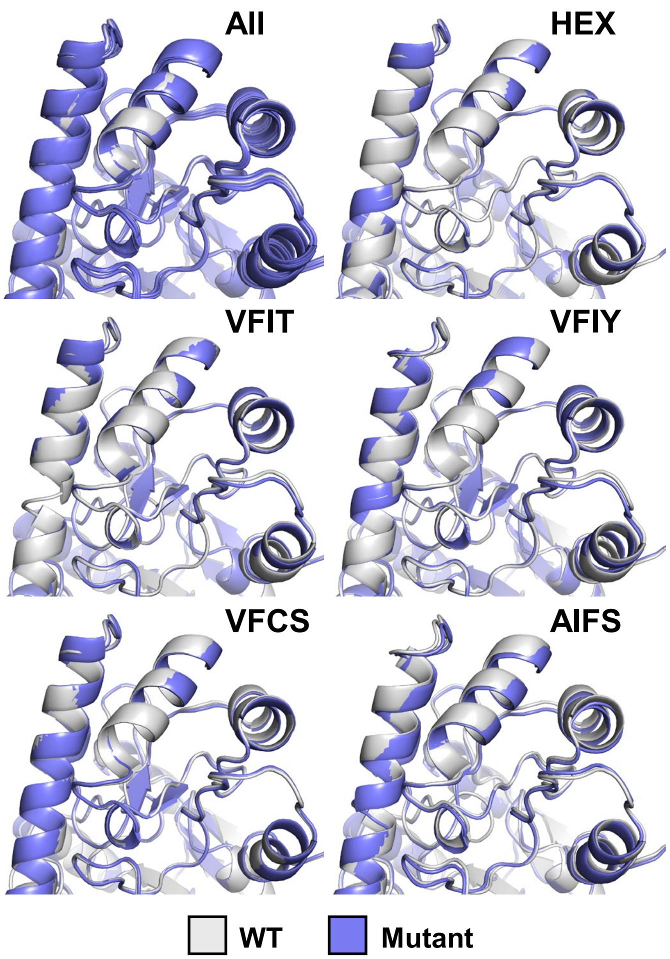

Supplementary Figure 7. Crystal structures of AAT variants in the maleate-bound form. Overlay of crystal structures shows that all six variants adopt nearly identical closed conformations in the presence of inhibitor.

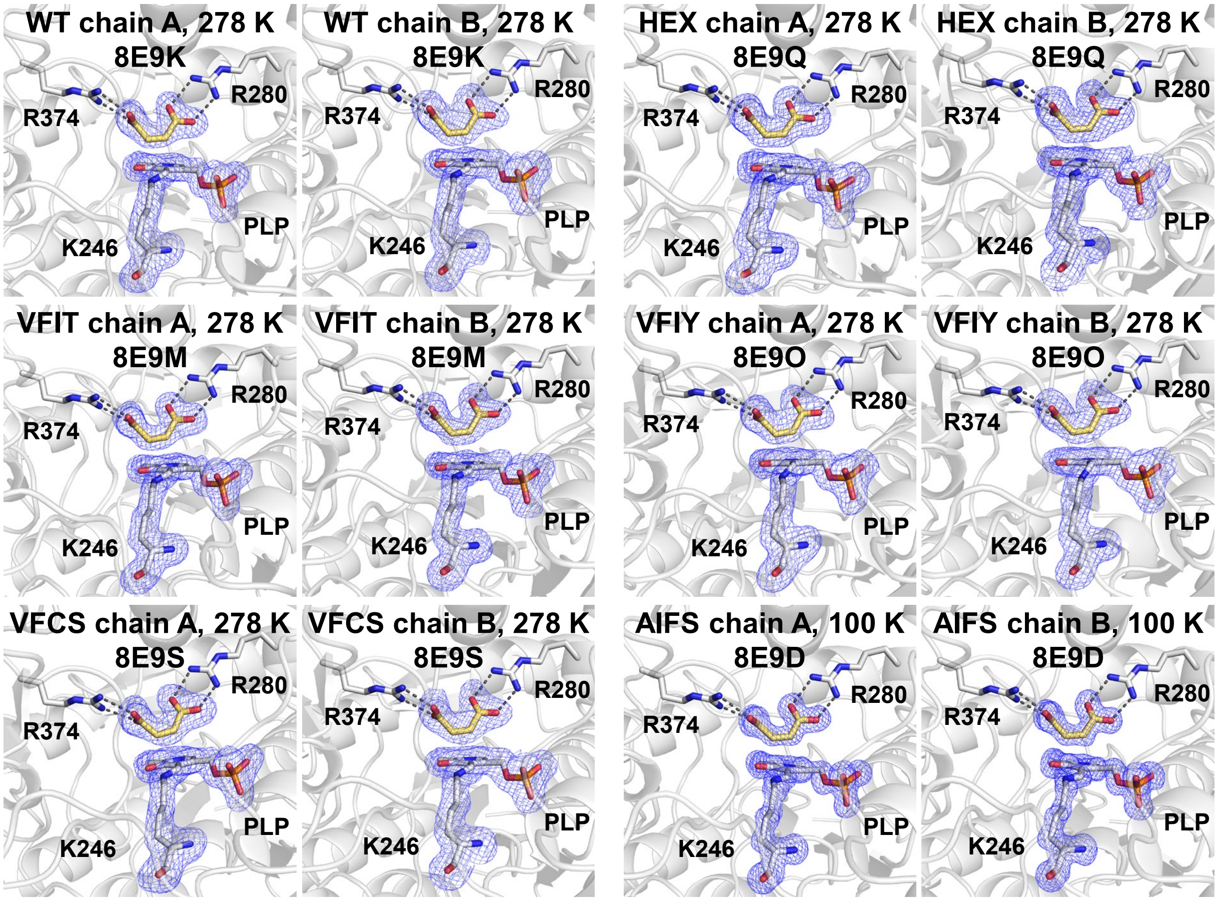

Supplementary Figure 8. Crystallographic evidence of the presence of bound maleate for all AAT variants. Presence of bound maleate (yellow), in both chain A and chain B of the enzyme models at various temperatures, is confirmed by an omit map (3.0σ, blue). The electron density supports a binding mode in which maleate forms hydrogen bonds (dashed lines) to both R280 and R374. In all cases, enzymes adopt the internal aldimine form where the catalytic K246 residue forms a Schiff base with the pyridoxal 5'-phosphate (PLP) cofactor. PDB IDs for all crystal structures are indicated.

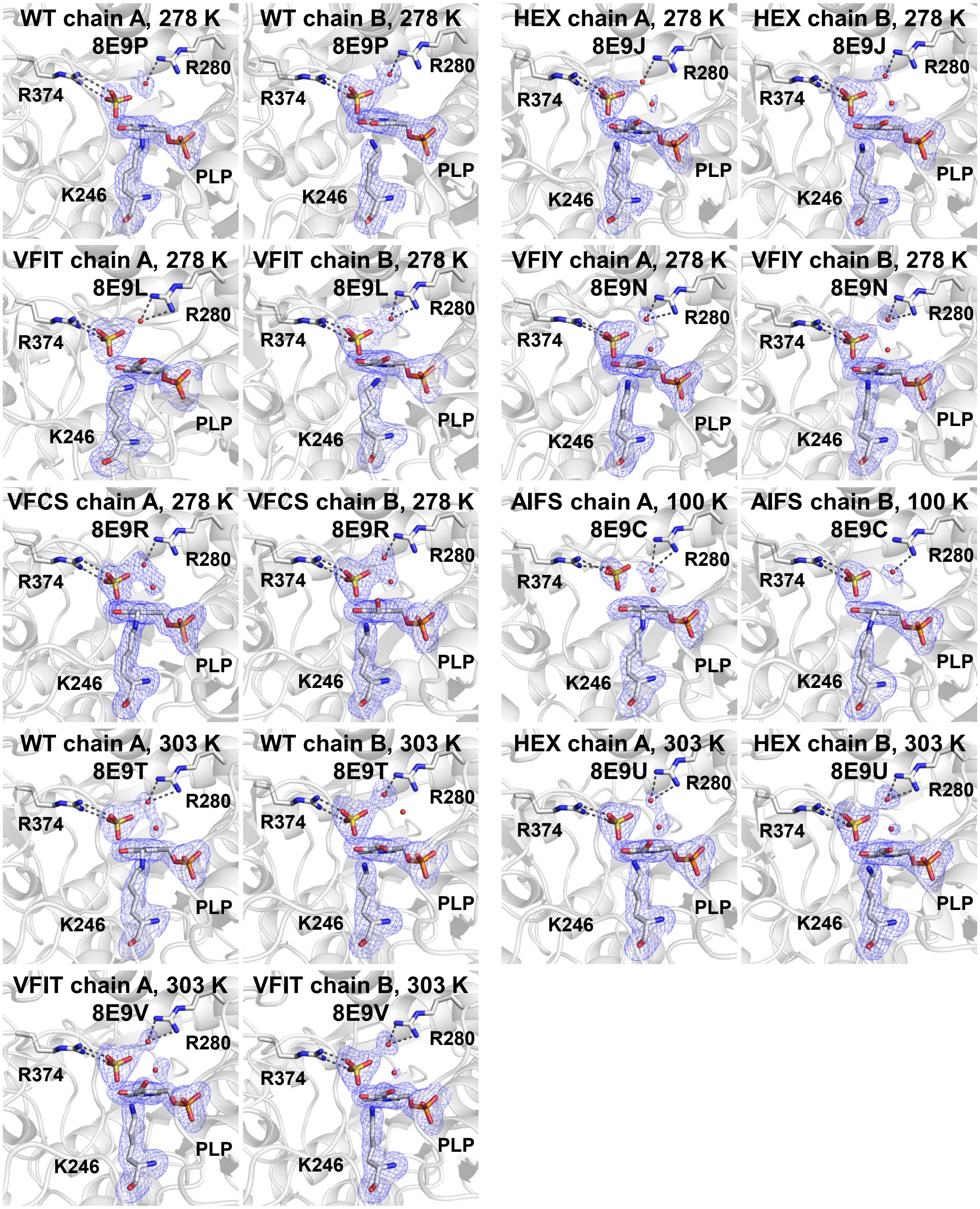

Supplementary Figure 9. Crystallographic evidence of maleate removal by crystal soaking. Removal of bound maleate, in both chain A and chain B of the enzyme models at various temperatures, is confirmed by an omit map (3.0σ, blue). The electron density supports replacement of maleate by a sulfate ion from the crystallization buffer and one or more water molecules (red spheres), which form electrostatic interactions or hydrogen bonds (dashed lines) to R280 or R374. The crystal soaking procedure to remove maleate also causes the loss of the covalent bond between the catalytic K246 residue and pyridoxal 5'-phosphate (PLP) cofactor in one or both subunits. PDB IDs for all crystal structures are indicated.

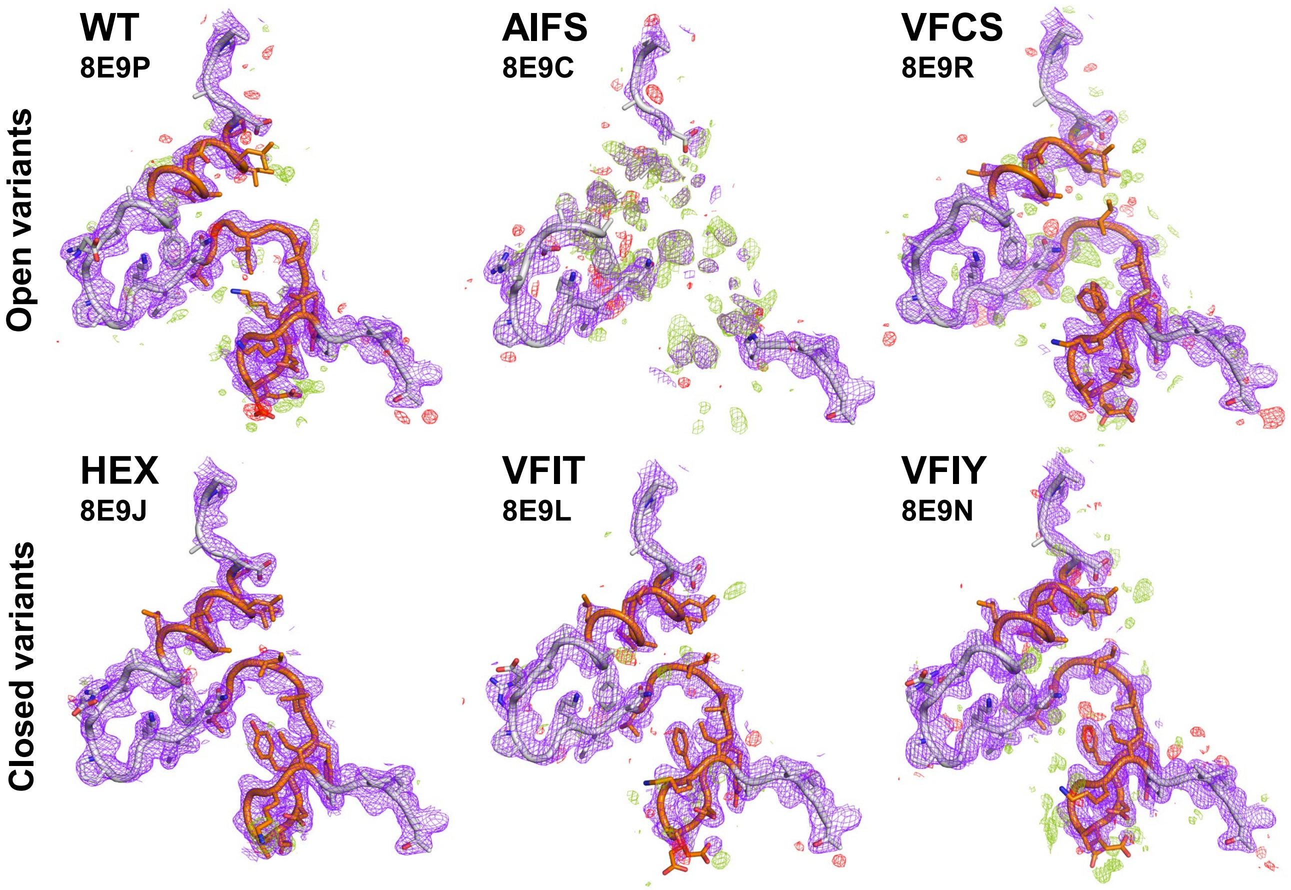

Supplementary Figure 10. Conformational heterogeneity in $\text{Open}_{\text{High}}$ library mutant AIFS. Electronic density of Chain A residues Ala8–Thr47 in all AAT variants in their ligand-free forms is shown (PDB IDs are indicated), with 2mFo-DFc electron density maps contoured at 1 σ (blue mesh) and mFo-DFc difference density maps contoured at ± 3 σ (green/red mesh). There is much heterogeneity in electron density around residues Pro12–Leu19 and Leu31–Thr43 (orange) in AIFS but not other variants.

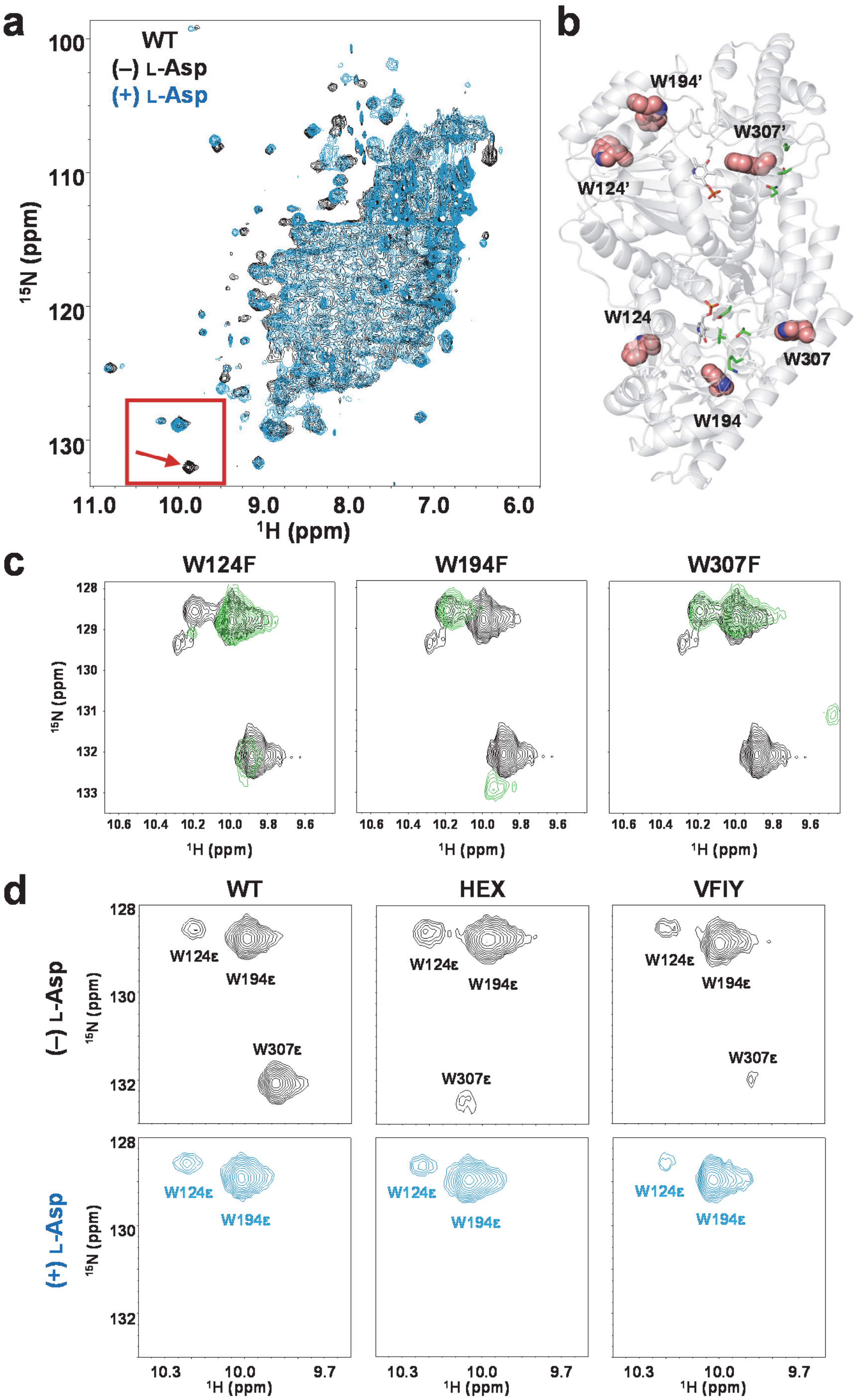

Supplementary Figure 11. ^{1}H-^{15}N HSQC spectra of selected AAT variants. (a) Addition of 5 mM donor substrate to wild-type (WT) AAT causes the shifting of peaks throughout the spectrum and disappearance of a peak (indicated by arrow) in the Trp side-chain region (boxed). (b) Trp residues whose side-chain amides yield peaks in the Trp side-chain region are shown as spheres (salmon). The PLP cofactor bound at the active site and designed residues (V35, K37, T43, N64) are shown as white and green sticks, respectively. (c) Overlay of WT spectrum (black) on those of single point mutants (green) allowed assignment of Trp indole NH peaks. All spectra were measured in the absence of substrate. (d) The side-chain peak of W307 disappears when 5 mM L-aspartate is added. In HEX and the closed library mutant VFIY, this peak is already substantially broadened in the absence of substrate, providing initial evidence that the conformational equilibrium in these variants favours the closed state.

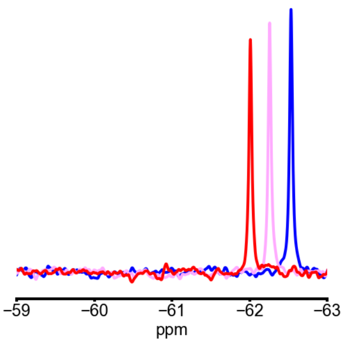

Supplementary Figure 12. ^{19}F NMR spectra of 4-trifluoromethyl-L-phenylalanine at various temperatures. Blue, purple, and red lines correspond to spectra at 278, 293, and 308 K, respectively.

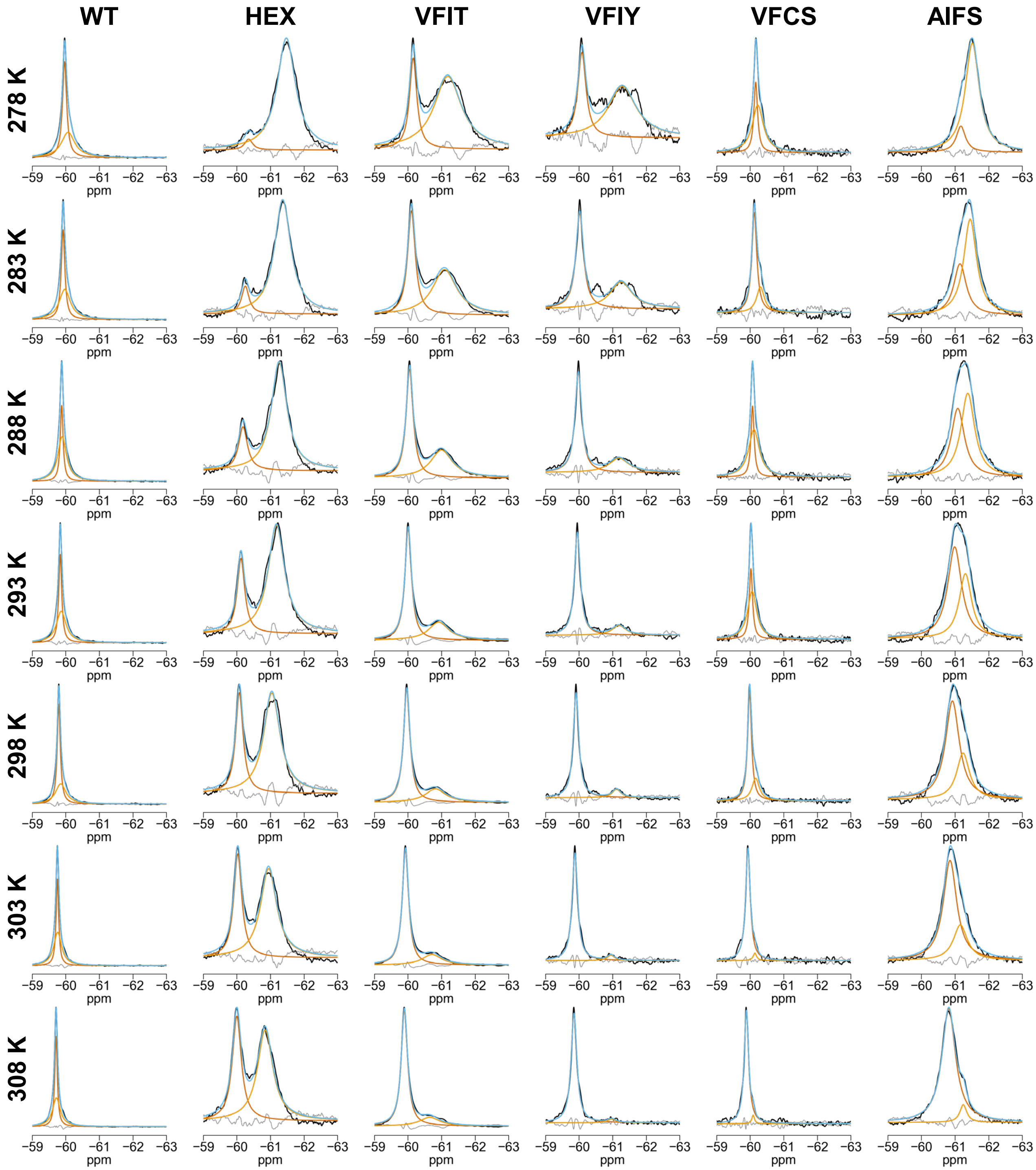

Supplementary Figure 13. Deconvolution of ^{19}F NMR spectra at various temperatures. The Lorentzian function was used to fit two peaks (light and dark orange) to the experimental NMR spectra (black) of various AATs. Fitted spectra and residuals are colored blue and grey, respectively. For WT and VFCS, deconvolution of spectra as two separate peaks did not yield meaningful results.

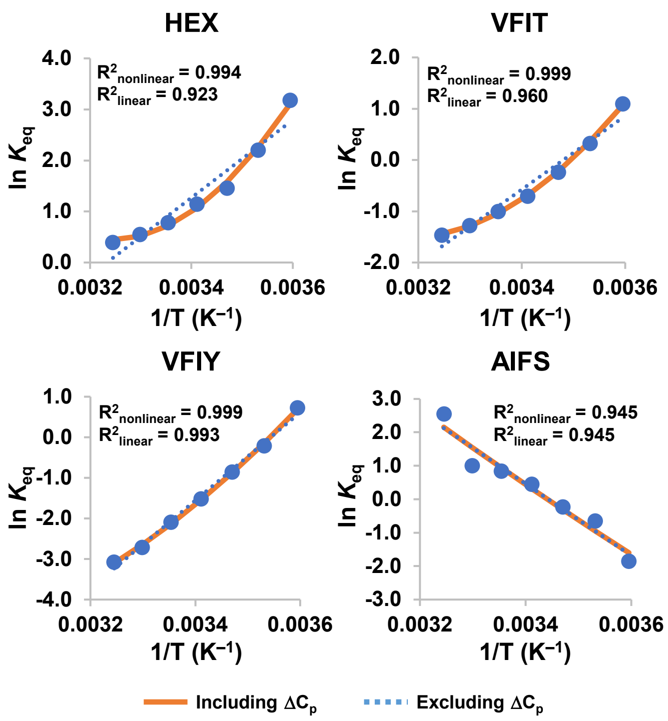

Supplementary Figure 14. Thermodynamic analysis of conformational exchange. The van't Hoff equation including (nonlinear) or excluding (linear) changes to heat capacity (ΔC_p) was used to fit equilibrium constants as a function of temperature. Coefficients of determination (R^2) close to unity for HEX, VFIT, VFIY, and AIFS confirm that these proteins are undergoing exchange. For HEX, VFIT, and VFIY, nonlinear fitting improves R^2 values, which is not the case for AIFS. For conformational exchange of AIFS between alternate open conformations (linear fit), we calculated ΔG, ΔH, and ΔS values (278 K) of 0.83 kcal mol^{-1}, 21.4 kcal mol^{-1}, and 0.074 kcal mol^{-1} K^{-1}, respectively.

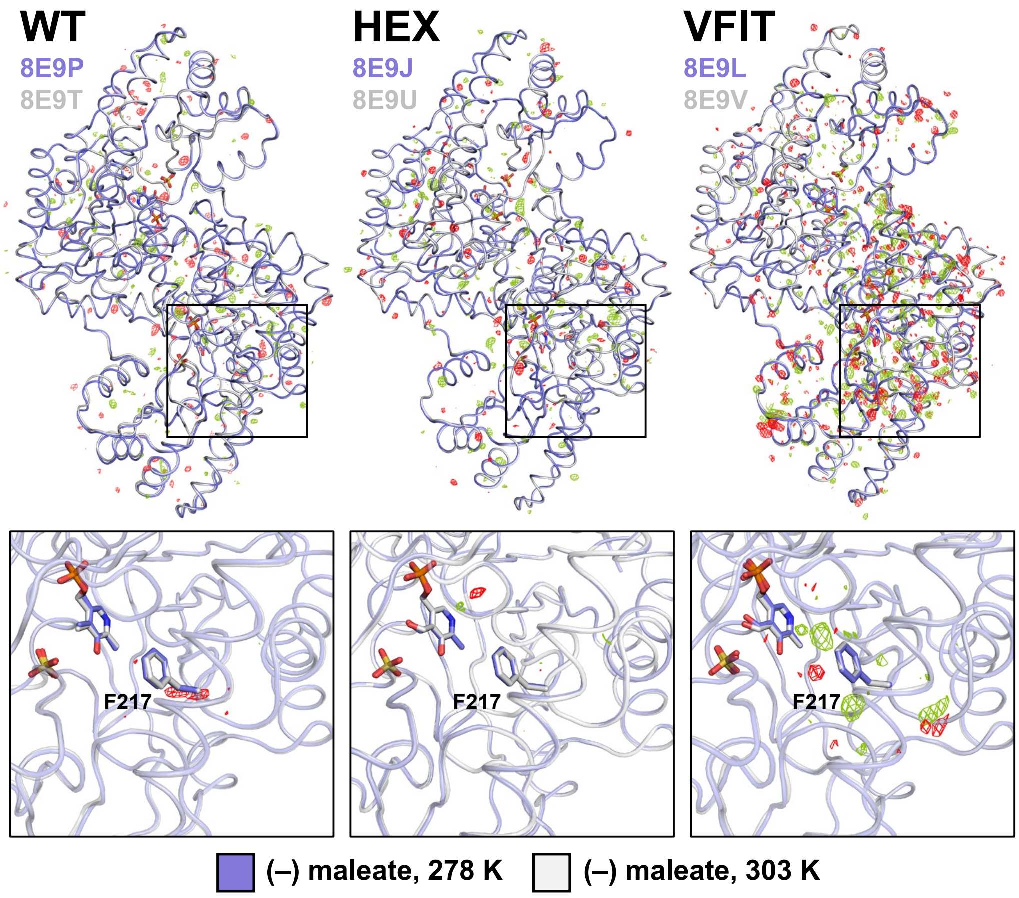

Supplementary Figure 15. Fo-Fo difference maps reveal increased local conformational changes in VFIT when temperature of crystal is increased from 278 K to 303 K. Top: Isomorphous Fo-Fo difference electron density map contoured at ± 3 σ (green/red mesh) for the 303 K dataset (white) minus the 278 K dataset (blue) in the absence of maleate. PDB IDs for crystal structures are indicated in blue (278 K) and light grey (303 K). Bottom: Difference density (± 3 σ) within a 5 Å radius of the F217 residue (sticks) where the 4-trifluoromethyl-L-phenylalanine amino acid was incorporated to enable multitemperature ^{19}F NMR spectroscopy. There is more difference density throughout chain A in the VFIT data set, even though the backbone conformation in crystal does not change substantially upon heating. The pyridoxal 5'-phosphate cofactor and sulfate ion that occupies the active site in the absence of maleate are shown as sticks. In all cases, isomorphous difference maps were generated using high- and low-resolution cut-offs of 2.31 and 20.0 Å, respectively.

```
WT      MAHHHHHHVGTFENITAAPADPILGLADLFRADERPGKINLGIGVYKDETGKTPVLTSVK  60
HEX     MAHHHHHHVGTFENITAAPADPILGLADLFRADERPGKINLGIGLYYDETGKIPVLTSVK  60
VFIT    MAHHHHHHVGTFENITAAPADPILGLADLFRADERPGKINLGIGVYFDETGKIPVLTSVK  60
VFIY    MAHHHHHHVGTFENITAAPADPILGLADLFRADERPGKINLGIGVYFDETGKIPVLTSVK  60
VFCS    MAHHHHHHVGTFENITAAPADPILGLADLFRADERPGKINLGIGVYFDETGKCPVLTSVK  60
AIFS    MAHHHHHHVGTFENITAAPADPILGLADLFRADERPGKINLGIGAYIDETGKFPVLTSVK  60
        ******************************************** * ***** *******

WT      KAEQYLLENETTKNYLGIDGIPEFGRCTQELLFGKGSALINDKRARTAQTPGGTGALRVA  120
HEX     KAEQYLLENETTKLYLGIDGIPEFGRCTQELLFGKGSALINDKRARTAQTPGGSGALRVA  120
VFIT    KAEQYLLENETTKTYLGIDGIPEFGRCTQELLFGKGSALINDKRARTAQTPGGTGALRVA  120
VFIY    KAEQYLLENETTKYYLGIDGIPEFGRCTQELLFGKGSALINDKRARTAQTPGGTGALRVA  120
VFCS    KAEQYLLENETTKSYLGIDGIPEFGRCTQELLFGKGSALINDKRARTAQTPGGTGALRVA  120
AIFS    KAEQYLLENETTKSYLGIDGIPEFGRCTQELLFGKGSALINDKRARTAQTPGGTGALRVA  120
        ************* ***************************************:******

WT      ADFLAKNTSVKRVWVSNPSWPNHKSVFNSAGLEVREYAYYDAENHTLDFDALINSLNEAQ  180
HEX     ADFLAKNTSVKRVWVSNPSWPNHKSVFNSAGLEVREYAYYDAENHTLDFDALINSLNEAQ  180
VFIT    ADFLAKNTSVKRVWVSNPSWPNHKSVFNSAGLEVREYAYYDAENHTLDFDALINSLNEAQ  180
VFIY    ADFLAKNTSVKRVWVSNPSWPNHKSVFNSAGLEVREYAYYDAENHTLDFDALINSLNEAQ  180
VFCS    ADFLAKNTSVKRVWVSNPSWPNHKSVFNSAGLEVREYAYYDAENHTLDFDALINSLNEAQ  180
AIFS    ADFLAKNTSVKRVWVSNPSWPNHKSVFNSAGLEVREYAYYDAENHTLDFDALINSLNEAQ  180
        ************************************************************

WT      AGDVVLFHGCCHNPTGIDPTLEQWQTLAQLSVEKGWLPLFDFAYQGFARGLEEDAEGLRA  240
HEX     AGDVVLFHGCCHNPTGIDPTLEQWQTLAQLSVEKGWLPLFDFAYQGFARGLEEDAEGLRA  240
VFIT    AGDVVLFHGCCHNPTGIDPTLEQWQTLAQLSVEKGWLPLFDFAYQGFARGLEEDAEGLRA  240
VFIY    AGDVVLFHGCCHNPTGIDPTLEQWQTLAQLSVEKGWLPLFDFAYQGFARGLEEDAEGLRA  240
VFCS    AGDVVLFHGCCHNPTGIDPTLEQWQTLAQLSVEKGWLPLFDFAYQGFARGLEEDAEGLRA  240
AIFS    AGDVVLFHGCCHNPTGIDPTLEQWQTLAQLSVEKGWLPLFDFAYQGFARGLEEDAEGLRA  240
        ************************************************************

WT      FAAMHKELIVASSYSKNFGLYNERVGACTLVAADSETVDRAFSQMKAAIRANYSNPPAHG  300
HEX     FAAMHKELIVASSYSKNFGLYNERVGACTLVAADSETVDRAFSQMKAAIRANYSSPPAHG  300
VFIT    FAAMHKELIVASSYSKNFGLYNERVGACTLVAADSETVDRAFSQMKAAIRANYSNPPAHG  300
VFIY    FAAMHKELIVASSYSKNFGLYNERVGACTLVAADSETVDRAFSQMKAAIRANYSNPPAHG  300
VFCS    FAAMHKELIVASSYSKNFGLYNERVGACTLVAADSETVDRAFSQMKAAIRANYSNPPAHG  300
AIFS    FAAMHKELIVASSYSKNFGLYNERVGACTLVAADSETVDRAFSQMKAAIRANYSNPPAHG  300
        ******************************************************.*****

WT      ASVVATILSNDALRAIWEQELTDMRQRIQRMRQLFVNTLQEKGANRDFSFIIKQNGMFSF  360
HEX     ASVVATILSNDALRAIWEQELTDMRQRIQRMRQLFVNTLQEKGANRDFSFIIKQNGMFSF  360
VFIT    ASVVATILSNDALRAIWEQELTDMRQRIQRMRQLFVNTLQEKGANRDFSFIIKQNGMFSF  360
VFIY    ASVVATILSNDALRAIWEQELTDMRQRIQRMRQLFVNTLQEKGANRDFSFIIKQNGMFSF  360
VFCS    ASVVATILSNDALRAIWEQELTDMRQRIQRMRQLFVNTLQEKGANRDFSFIIKQNGMFSF  360
AIFS    ASVVATILSNDALRAIWEQELTDMRQRIQRMRQLFVNTLQEKGANRDFSFIIKQNGMFSF  360
        ************************************************************

WT      SGLTKEQVLRLREEFGVYAVASGRVNVAGMTPDNMAPLCEAIVAVL   406
HEX     SGLTKEQVLRLREEFGVYAVASGRVNVAGMTPDNMAPLCEAIVAVL   406
VFIT    SGLTKEQVLRLREEFGVYAVASGRVNVAGMTPDNMAPLCEAIVAVL   406
VFIY    SGLTKEQVLRLREEFGVYAVASGRVNVAGMTPDNMAPLCEAIVAVL   406
VFCS    SGLTKEQVLRLREEFGVYAVASGRVNVAGMTPDNMAPLCEAIVAVL   406
AIFS    SGLTKEQVLRLREEFGVYAVASGRVNVAGMTPDNMAPLCEAIVAVL   406
        **********************************************
```

Supplementary Figure 16. Multiple sequence alignment of AAT variants.
